# Medroxyprogesterone acetate inhibits wound closure of human endometrial epithelial cells and stromal fibroblasts in vitro

**DOI:** 10.1038/s41598-021-02681-6

**Published:** 2021-12-01

**Authors:** Mickey V. Patel, Marta Rodriguez-Garcia, Zheng Shen, Charles R. Wira

**Affiliations:** 1 Department of Microbiology and Immunology, Geisel School of Medicine at Dartmouth, Lebanon, NH 03756 USA; 2 Department of Immunology, Tufts University School of Medicine, Boston, MA USA

**Keywords:** Mucosal immunology, Cell migration

## Abstract

Mucosal integrity in the endometrium is essential for immune protection. Since breaches or injury to the epithelial barrier exposes underlying tissue and is hypothesized to increase infection risk, we determined whether endogenous progesterone or three exogenous progestins (medroxyprogesterone acetate (MPA), norethindrone (NET), and levonorgestrel (LNG)) used by women as contraceptives interfere with wound closure of endometrial epithelial cells and fibroblasts in vitro. Progesterone and LNG had no inhibitory effect on wound closure by either epithelial cells or fibroblasts. MPA significantly impaired wound closure in both cell types and delayed the reestablishment of transepithelial resistance by epithelial cells. In contrast to MPA, NET selectively decreased wound closure by stromal fibroblasts but not epithelial cells. Following epithelial injury, MPA but not LNG or NET, blocked the injury-induced upregulation of HBD2, a broad-spectrum antimicrobial implicated in wound healing, but had no effect on the secretion of RANTES, CCL20 and SDF-1α. This study demonstrates that, unlike progesterone and LNG, MPA and NET may interfere with wound closure following injury in the endometrium, potentially conferring a higher risk of pathogen transmission. Our findings highlight the importance of evaluating progestins for their impact on wound repair at mucosal surfaces.

## Introduction

More than 1 million sexually transmitted infections (STIs) are acquired every day with annual estimates of 376 million new infections of chlamydia, gonorrhea, syphilis, trichomoniasis and HIV^1^. A key component of protection against STIs in women is the mucosal surface of the female reproductive tract (FRT) that acts as both a physical and immunological barrier to pathogens. Breakdown of barrier function can allow for increased movement of pathogens from the luminal environment into the underlying tissue. Two major cellular components of the mucosal barrier are epithelial cells and fibroblasts. In the FRT, the upper tract (endocervix, endometrium, Fallopian tubes) is covered by a single layer of columnar epithelial cells, distinct from the multilayered squamous epithelium in the lower FRT (vagina, ectocervix). Held together by tight junctions, columnar epithelial cells in the endometrium function as a physical barrier and along with underlying stromal cells, serve as essential sources of antimicrobials that interfere with STI entry^2,3^. Disruptions to the mucosal barrier in the upper FRT occur in multiple ways. Ulcerations, endometritis, menstruation, parturition, and cancer all can contribute to a weakening of the mucosal barrier in the upper FRT. Furthermore, STIs such as HIV, HSV-2, and *Chlamydia,* which enter the via the lower FRT, can directly degrade epithelial barrier function leading to enhanced transmission^4–6^. Rapid recovery of the upper FRT mucosal surface from these disruptions is necessary for reproductive health and survival.

Synthetic progestins such as medroxyprogesterone acetate (MPA), norethindrone (NET) and levonorgestrel (LNG) are administered to millions of women for multiple reasons including contraception, menopausal hormone replacement therapy, endometriosis, and cancer^7–9^. As contraceptives, MPA and NET are predominantly used in injectable form, while LNG is administered in intra-uterine devices (IUDs)^10,11^. MPA, a commonly used contraceptive in Sub-Saharan Africa, is linked to increased risk of HIV transmission^12^. This is supported by multiple in vitro studies that demonstrate MPA can modulate immune function in the FRT^13–16^. In contrast, NET and LNG do not appear to increase the risk of HIV transmission^17–19^.

Despite their use by millions of women, the effects of progestins on mucosal integrity in the upper FRT is unclear, with the majority of research focusing on the lower FRT. Several studies have reported that MPA causes a thinning of the ectocervical epithelium and alters the distribution of HIV target cells, both of which are consistent with increases in HIV acquisition^20,21^ and other STIs^22–24^. Others have shown that markers of mucosal integrity and epithelial repair proteins are suppressed, and epithelial injury markers increased, in women using MPA^25,26^, while MPA exposure in vitro results in decreased barrier integrity and function of vaginal epithelial cells^27,28^. MPA also increases penetration of HIV virions into the cervical columnar epithelium of rhesus macaques^29^. Together, these studies demonstrate that MPA can weaken the mucosal barrier in the lower FRT. However, whether progestins affect the ability of the mucosal barrier to recover from injury, or modulate wound closure in the upper FRT, remains unclear.

Since progestins have not been considered as confounding factors in wound closure following injury in the FRT, we evaluated the effects of MPA, NET and LNG on wound closure by endometrial epithelial cells and stromal fibroblasts in vitro. We report that MPA inhibits the wound closure of primary endometrial epithelial cells and stromal fibroblasts in response to injury. In contrast, NET selectively inhibits wound closure by fibroblasts but not epithelial cells while progesterone and LNG have no effect on either cell type. This suggests that specific progestins, unlike endogenous progesterone, can selectively compromise wound closure and thus recovery of mucosal barrier function following injury in the upper FRT, increasing the potential for pathogens to enter the underlying stromal tissue and compromise reproductive health. Our findings highlight the importance of identifying synthetic steroid hormones that will not compromise immune protection by interfering with mucosal barrier function in the upper FRT.

## Materials and methods

### Ethics committee

All investigations were conducted according to the principles expressed in the Declaration of Helsinki and carried out with the approval from the Committee for the Protection of Human Subjects (CPHS), Dartmouth-Hitchcock Medical Center, and with written informed consent obtained from the patients before surgery.

### Study subjects

Human endometrial tissues were obtained from women undergoing hysterectomy surgery at Dartmouth-Hitchcock Medical Center (Lebanon, NH). Women were aged between 28 and 60 years old. All tissues used in this study were distal to the sites of pathology and were determined to be unaffected with disease upon inspection by a pathologist.

### Isolation of endometrial epithelial cells and fibroblasts

Endometrial tissues were minced under sterile conditions into 1 to 2 mm fragments and enzymatically digested using a mixture consisting of 0.05% collagenase type IV (Sigma-Aldrich, St Louis, MO) and 0.01% DNAse (Worthington Biochemical, Lakewood, NJ) for up to 1 h at 37 °C. After enzymatic digestion, cells were dispersed through a 250 µm mesh screen (Small Parts, Miami Lakes, FL), washed, and resuspended in Hank’s Balanced Salt Solution (HBSS) (Thermo Fisher, Logan, UT). Epithelial cell sheets (consisting of glandular and luminal epithelial cells in variable proportions) were separated from stromal fibroblasts by sequential filtration through a 40 and 20 µm nylon mesh filter (Small Parts, Miami Lakes, FL). While not determined in this study, the ratio of glandular to luminal epithelial cells varies from patient to patient since our population included both pre- and postmenopausal women between the ages of 28–60 years old. Epithelial sheets and glands were retained on the surface of both filters and recovered by rinsing and backwashing the filter with DMEM/F12 (Thermo Fisher), which was then centrifuged (500×*g,* 10 min), and analyzed for cell number and viability^30,31^. The stromal fraction containing fibroblasts passed through the filters and was collected as part of the filtrate^30,31^.

### Transwell epithelial cell culture

Epithelial cells were plated in 0.4 μm 24-well plate Matrigel-coated transwell inserts (Corning Life Sciences, Tewksbury, MA) and grown in defined media consisting of DMEM/F12 (Thermo Fisher) supplemented with NuSerum (Corning Life Sciences), Hyclone Defined FBS (GE Life Sciences, Logan, UT), Penicillin–Streptomycin (Thermo Fisher), l-Glutamine (Thermo Fisher) and HEPES (GE Life Sciences) as previously described by us^30,31^. Growth on transwell inserts allows for the formation of distinct apical and basolateral compartments^30,31^. Cells were grown to confluence and allowed to polarize as determined by transepithelial resistance (TER) of greater than 1000 ohms per insert^30,31^. TER was measured using an EVOM electrode and Voltohmmeter (World Precision Instruments, Sarasota, FL). Only polarized preparations of epithelial cells above 1000 ohms were used in this study. 24 h before progestin treatment, cells were transferred to stripped media.

### 96-well epithelial cell culture

Epithelial cells were plated in Matrigel-coated 96-well image-locked plates from Essen Biosciences (Ann Arbor, MI) and grown to confluence^31^. 24 h before progestin treatment, cells were transferred to stripped media.

### Stromal fibroblast cell culture

Stromal cell suspensions from the endometrium were cultured in T-75 flasks (Corning Life Sciences) in stromal defined media consisting of DMEM/F12 (Thermo Fisher) supplemented with Hyclone Defined FBS (GE Life Sciences), Penicillin–Streptomycin (Thermo Fisher), l-Glutamine (Thermo Fisher) and HEPES (GE Life Sciences). Once cells reached confluence, they were passaged using trypsin–EDTA and grown to confluence again. After 3–4 passages a purified population of stromal fibroblasts remained, characterized as vimentin + , CD90 + , and CD45- as previously shown^3^. Stromal fibroblasts were then plated in either (i) 24-well plates (USA Scientific, Ocala, FL) or (ii) 96-well image-locked plates (Essen Biosciences); and grown for 48 h in stripped media prior to progestin or hormone treatment.

### Hormone treatment

Medroxyprogesterone acetate (MPA), Norethindrone Acetate (NET), Levonorgestrel (LNG), or progesterone (P) (All Sigma-Aldrich, St. Louis, MO) were dissolved in 100% ethanol for an initial concentration of 1000 µM, evaporated to dryness in a glass scintillation vial and resuspended to a concentration of 10 µM in stripped media. Stripped media was DMEM/F12 supplemented with charcoal–dextran stripped FBS (Gemini Bio-Products, West Sacramento, CA), Penicillin–Streptomycin (Thermo Fisher), l-Glutamine (Thermo Fisher), and HEPES (GE Life Sciences). Further dilutions were made to achieve final working concentrations of 0.1 to 100 nM. As a control, an equivalent amount of ethanol without hormone was evaporated to dryness prior to media addition.

### Scratch assay

The scratch assay was performed using two experimental approaches as previously described by us^31^. (i) Transwell inserts (wound closure): confluent monolayers of endometrial epithelial cells in 0.4 µm transwell inserts and stromal fibroblasts in 24-well plates were scratched using a 20 μl pipette tip attached to a wooden handle^31^. This allows for scratches of consistent width and length. Scratch width and TER were measured immediately afterwards and at regular intervals as noted in the results. Marked plates were used to ensure that scratches were measured at the same location at different time points. Wound healing was measured as percentage of wound closure at 24- and 48-h post-scratch compared to initial wound width. (ii) 96-well plates (relative wound density): confluent monolayers of endometrial epithelial cells and stromal fibroblasts grown to confluence in 96-well image-locked plates were scratched using a WoundMaker from Essen Biosciences which allows for simultaneous injury of up to 96 wells (see below for further details). Wound healing was analyzed using the IncuCyte ZOOM (Essen Biosciences) with the Scratch Wound Cell Migration Software Module (Essen Biosciences), which allows for real-time analysis of wound healing. Wound healing was assessed as relative wound density (cellular density in the wound area relative to the cellular density outside the wound area). Please see http://www.essenbioscience.com/en/applications/live-cell-assays/scratch-wound-cell-migration-invasion/ for further details.

### Dextran flux and cell proliferation assays

FITC-Dextran (4 kDa) (Sigma-Aldrich) was dissolved in stripped media and added to the apical compartment of transwell inserts 48 h after injury at a final concentration of 100 µg/ml for 6 h. After 6 h, 100 µl of media from the basolateral compartment was recovered and the amount of FITC-Dextran assessed using a luminometer.

Cell proliferation was determined using the CellTiter Aqueous One cell proliferation assay (Promega Corporation, Madison, WI) according to the manufacturer’s instructions. Briefly, the CellTiter assay is a colorimetric method for determining the number of viable cells in proliferation assays. It measures the breakdown of a tetrazolium compound (MTS) and an electron coupling reagent (PES) into formazan in the presence of proliferating cells. The quantity of formazan product as measured by the amount of 490 nm absorbance is directly proportional to the number of living cells in culture. For further details: https://www.promega.com/products/cell-health-assays/cell-viability-and-cytotoxicity-assays/celltiter-96-aqueous-one-solution-cell-proliferation-assay-_mts_/.

### Cytokine secretion

Concentrations of HBD2, CCL20, RANTES, and SDF-1α in cell culture supernatants were determined using a custom microsphere multiplex assay developed by our group^32^. The inter-assay coefficients of variation (CV) were 6.1% (HBD2), 8.5% (CCL20), 5.9% (RANTES), and 7.4% (SDF-1α).

### Statistical analysis

GraphPad Prism 5.0 (GraphPad Software, La Jolla, CA) was used for data analysis with *p* < 0.05 statistically significant. A Mann–Whitney test for non-matched samples or Wilcoxon matched-pairs signed rank test for matched samples, was used to compare non-matched and matched samples respectively. Comparison of three or more groups was performed using the Kruskal–Wallis test for non-matched samples and Friedman test for matched samples, followed by Dunns-post test for multiple comparison correction^31^.

## Results

### MPA and NET but not LNG or progesterone inhibit wound closure of endometrial epithelial cells and stromal fibroblasts

**Figure 1:**
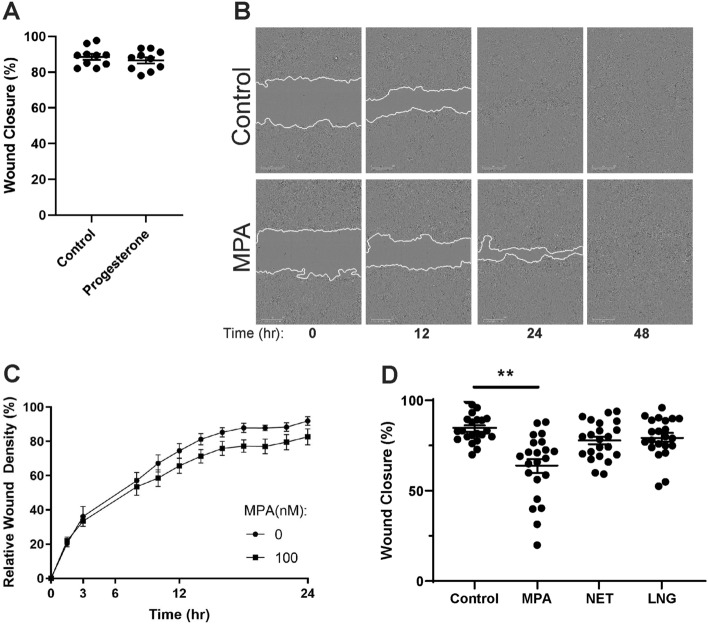
MPA inhibits wound closure by endometrial epithelial cells. **(A)** Wound closure of endometrial epithelial cells grown in transwell inserts at 24 h post-scratch following pre-treatment for 48 h with 100 nM progesterone. Each symbol represents a single individual (*n* = *10*). **(B)** Representative images of endometrial epithelial cells grown in 96-well plates scratched using the IncuCyte WoundMaker in the absence (control) or presence of MPA (100 nM) at 0, 12, 24, and 48 h at  × 10 magnification using the IncuCyte Zoom. Time of scratch is 0 h. The solid white line represents the epithelial wound front. **(C)** Relative wound density of endometrial epithelial cells grown in 96-well plates was measured at the indicated time points for 24 h post-scratch in the presence (100 nM) or absence of MPA. Data shown is from another representative patient than **(B)**. Each symbol represents the mean + /− SEM from a single timepoint. Each timepoint is the average of triplicate or quadruplicate wells. **(D)** Wound closure of endometrial epithelial cells grown in transwell inserts at 24 h post-scratch following pre-treatment with 100 nM MPA, NET, and LNG for 48 h prior to scratch. MPA, NET, LNG, and Progesterone were maintained in cell culture media at 100 nM following scratch. Each symbol represents a single individual (*n* = *22*).

Since progestins are used by millions of women worldwide^10^, and may be detrimental for barrier integrity^25^, we investigated whether in vitro wound closure of monolayers of endometrial epithelial cells and stromal fibroblasts is modulated by exposure to progestins. Our initial studies focused on the effects of the endogenous sex hormone progesterone, which undergoes cyclic changes across the menstrual cycle in premenopausal women. Progesterone receptor (PR) mRNA is expressed by endometrial epithelial cells grown in vitro (Supplementary Fig. 1). To examine the impact of progesterone on wound closure, endometrial epithelial cells were grown to confluence on transwell inserts and then exposed to progesterone (100 nM) for 48 h, followed by injury with a pipette tip^31^. Width of the wound was measured immediately following injury, and at 24 and 48 h post-injury during which progesterone was maintained in the culture media. As seen in Fig. 1A, there was no significant effect of progesterone on wound closure by endometrial epithelial cells compared to untreated controls 24 h post-injury, or at 48 h post-injury (data not shown).

Since progesterone had no effect on wound healing by endometrial epithelial cells, we investigated the effects of the synthetic progestins MPA, NET, and LNG. Monolayers of endometrial epithelial cells grown in 96-well plates were treated with MPA, NET, or LNG (100 nM) for 48 h prior to injury, followed by a further 48 h of exposure to progestin. During this period images of the epithelial monolayer were taken using an IncuCyte Zoom. As seen in representative scratch assays (Fig. 1B,C), relative wound density of epithelial monolayers treated with MPA was lower relative to untreated controls by approximately 10–15% at 12 and 24 h post-injury. Similarly, MPA significantly slowed wound closure by epithelial monolayers grown on transwell inserts 24 h post-injury, at which time wound closure in untreated epithelial monolayers was almost complete (Fig. 1D). In contrast, there was no significant effect of either NET or LNG on wound closure at 24 h (Fig. 1D). By 48 h post-injury, wound closure was complete under all conditions.

### MPA and NET inhibit restoration of endometrial epithelial cell barrier integrity following injury

**Figure 2:**
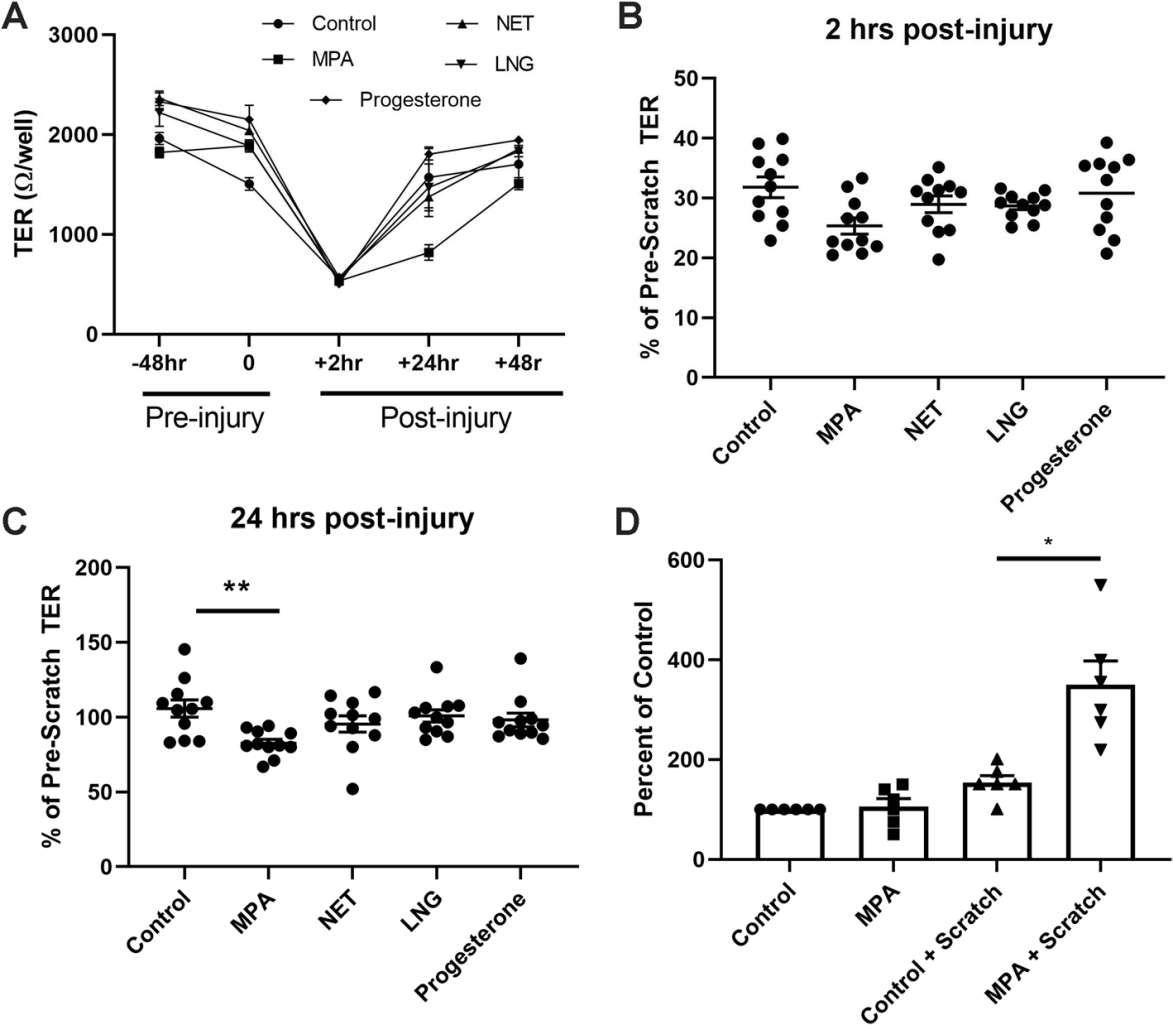
MPA decreases barrier integrity and increases paracellular permeability of endometrial epithelial cells treated with MPA. **(A)** Representative transepithelial resistance (TER) profile of endometrial epithelial cells grown in transwell inserts pre- and post-scratch during exposure to 100 nM MPA, NET, LNG, or Progesterone. Scratch was performed at 0 h. Each symbol represents the mean + /− SEM from a single time point. Data shown is from a representative patient. **(B)** TER of endometrial epithelial cells grown in transwell inserts 2 h and **(C)** 24 h following scratch treated with 100 nM MPA, NET, and LNG. Each symbol represents a single individual (*n* = *11*). **(D)** FITC-Dextran 4KDa flux assay 24 h post-scratch performed over 6 h. Cells were treated with MPA, NET, or LNG at 100 nM for 48 h prior to scratch, and maintained in cell culture media at 100 nM post-scratch. Bars represent mean + /− SEM. Each symbol represents a single individual (*n* = *6*). Values are normalized to the control unscratched condition which is set to 100. *p < 0.05; **p < 0.01.

A primary function of epithelial cells is to form a physical barrier that separates the internal and external environment and prevent entry of external pathogens^33^. A key component of the barrier function is permeability, which can be assessed by transepithelial resistance (TER). In parallel with our wound closure studies, we measured the effects of MPA, NET and LNG on TER following injury of endometrial epithelial monolayers grown on transwell inserts. As seen in a representative time course study (Fig. 2A), injury leads to a decrease in TER followed by a rapid recovery so that by 24–48 h TER has returned to pre-injury values. The immediate decrease in TER after injury occurs irrespective of pretreatment conditions and is consistent with the loss of cellular coverage leading to gaps in the monolayer and an inability to control movement across these regions. As seen in Fig. 2B, at 2 h post-injury TER decreased by at least 70–80% irrespective of treatment conditions relative to pre-scratch values. By 24 h post-injury, TER of control monolayers recovered to pre-injury values (Fig. 2C). In contrast, recovery of TER by monolayers treated with MPA was significantly delayed by 20–30% at 24 h post-injury compared to untreated controls (Fig. 2C). This is consistent with reduced wound closure at 24 h post-injury in MPA-treated monolayers. By 48 h post-injury, TER from MPA-treated monolayers had returned to pre-injury values (Fig. 2A). In contrast, there was no inhibitory effect of NET, LNG, or progesterone on recovery of TER following injury at any time point which is consistent with our findings that they do not compromise wound closure of endometrial epithelial cells.

### Increased paracellular flux across MPA-treated epithelial monolayers

To determine whether injury and/or MPA exposure affects paracellular movement across a monolayer of polarized epithelial cells, we used a dextran-flux assay that measures the movement of labelled dextran from the apical to basolateral compartment of transwell inserts. Following 24 h post-injury, epithelial cells were apically exposed to FITC-Dextran (4 kDa) for 6 h, after which media from the basolateral compartment was assessed for the presence of FITC-Dextran. As seen in Fig. 2D, in the absence of injury there was no significant difference in dextran flux from the apical to basolateral compartment of polarized epithelial monolayers between untreated (control) and MPA-treated (100 nM) cells. There was also no significant difference in dextran flux between untreated monolayers (control) in the absence of injury or following injury measured at 24 h post scratch. In contrast, compared to injured untreated epithelial monolayers, we found a significant increase in dextran flux across MPA-treated epithelial monolayers following injury. These results are consistent with the MPA-induced delays in closure and reduced TER observed at 24 h (Fig. 1).

### MPA and NET inhibit wound closure of endometrial fibroblasts

**Figure 3:**
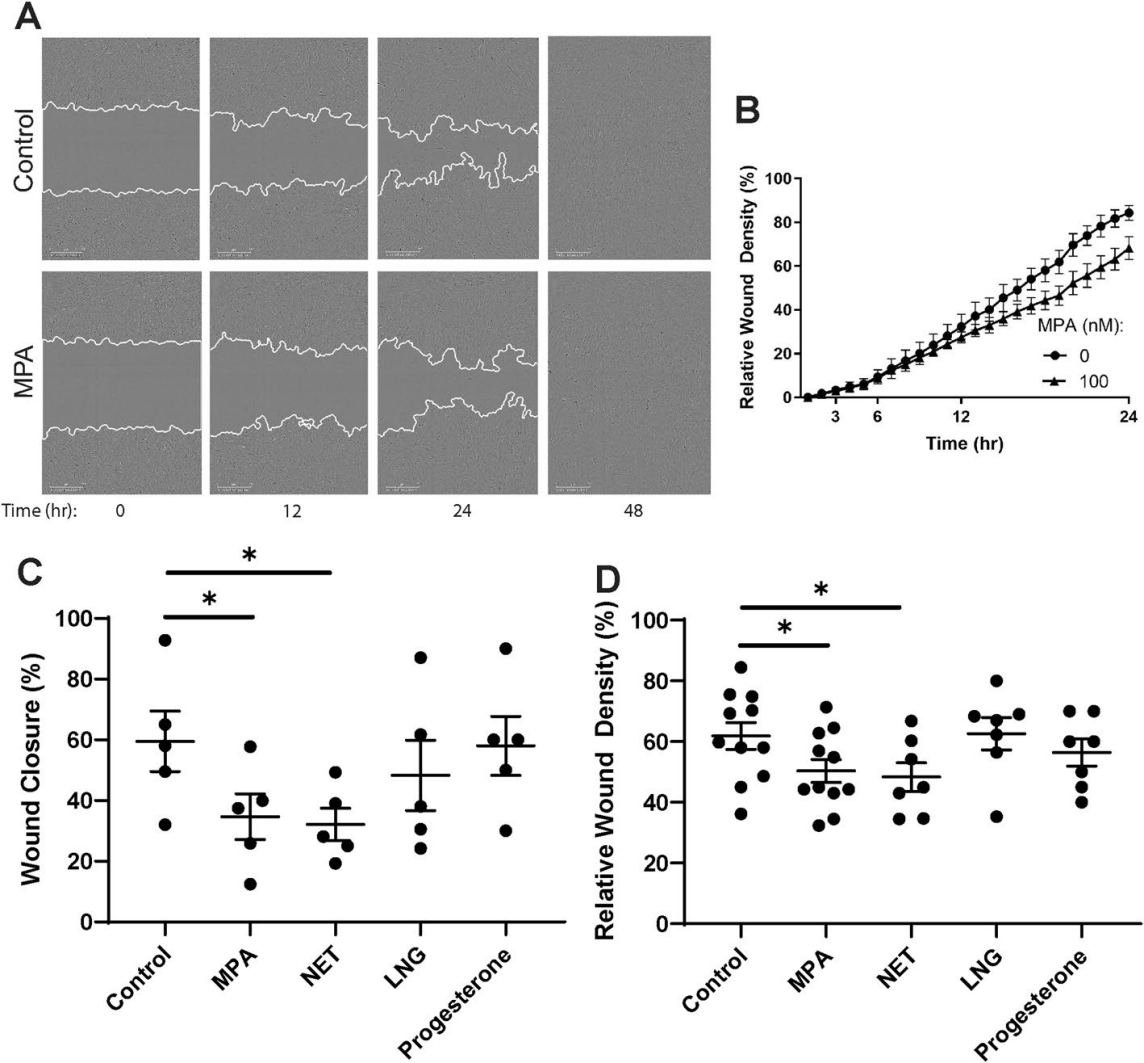
MPA and NET inhibit wound closure by endometrial stromal fibroblasts. **(A)** Representative images of endometrial stromal fibroblasts grown in 96-well plates scratched using an IncuCyte WoundMaker in the absence (control) or presence of MPA (100 nM) at 0, 12, 24, and 48 h at  × 10 magnification using the IncuCyte Zoom. Time of scratch is 0 h. The solid white line represents the fibroblast wound front. **(B)** Relative wound density of endometrial stromal fibroblasts grown in 96-well plates 24 h post-scratch in the presence (100 nM) or absence of MPA. Data shown is from a representative patient. Each symbol represents the mean + /− SEM from a single timepoint. **(C)** Wound closure of endometrial stromal fibroblasts grown in 24-well plates at 24 h post-scratch following treatment with 100 nM MPA, NET, LNG, or Progesterone for 48 h prior to scratch. MPA, NET, LNG, and Progesterone were maintained in cell culture media at 100 nM following scratch. Each symbol represents a single individual (*n* = *5*). (**D**) Relative wound density of endometrial stromal fibroblasts grown in 96-well plates 24 h post-scratch following treatment with 100 nM MPA, NET, LNG, or Progesterone. Each symbol represents a single individual (MPA *n* = *10*; NET *n* = *7*; LNG *n* = *7;* Progesterone *n* = *7*). *p < 0.05.

Recognizing that damage to the epithelium often extends into sub-epithelial tissues, we investigated the effect of progestins on wound healing of endometrial fibroblasts, which form a dense a layer immediately below the epithelial cells. Endometrial fibroblasts were grown to confluence and treated with MPA, NET, LNG, or progesterone (100 nM) for 48 h prior to and after injury. Similar to epithelial cells, endometrial fibroblasts express PR mRNA (Supplementary Fig. 1). As seen in representative assays (Fig. 3A,B), closure was delayed at 24 h with MPA-treated cells relative to control cells. MPA significantly slowed wound closure (Fig. 3C) and relative wound density (Fig. 3D) by approximately 15–20% at 24 h post-injury compared to untreated controls. Similarly, NET also significantly suppressed wound closure and relative wound density 24 h post-injury. In contrast, LNG and progesterone had no suppressive effect on wound closure by endometrial fibroblasts. By 48 h post-injury wound closure was complete under all conditions.

### MPA dose-dependently inhibits wound closure by endometrial epithelial cells and fibroblasts

**Figure 4:**
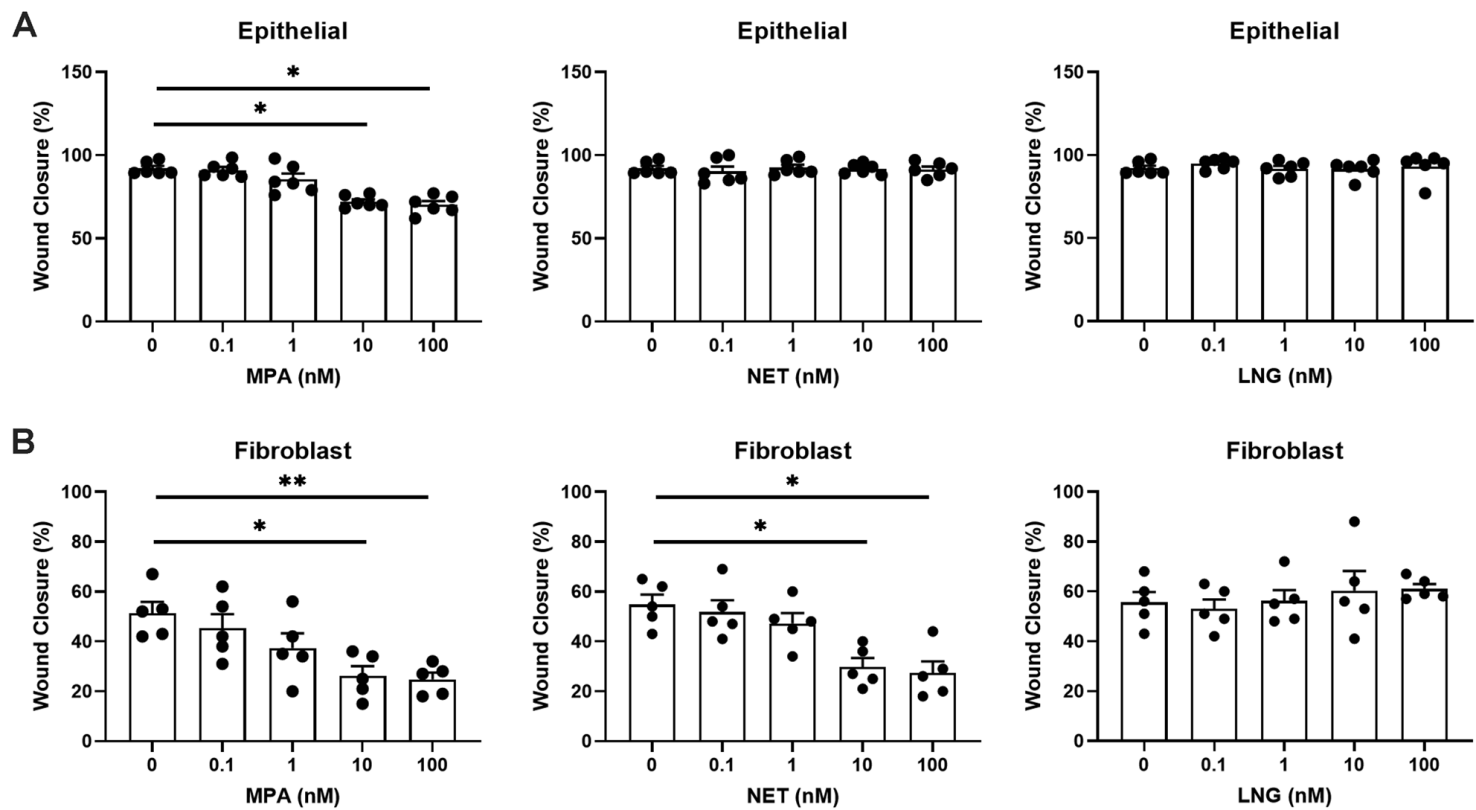
MPA and NET exhibit a dose-dependent suppression of wound closure in endometrial epithelial cells and stromal fibroblasts. **(A)** Wound closure by endometrial epithelial cells grown in transwell inserts and **(B)** wound closure by endometrial stromal fibroblasts grown in 24-well plates 24 h post-scratch following treatment with 0.1, 1, 10, and 100 nM MPA, NET, and LNG for 48 h prior to scratch. MPA, NET, and LNG were maintained in the cell culture media following scratch. Bars represent mean + /− SEM. Each symbol represents a single individual (epithelial cell *n* = *6*; stromal fibroblast *n* = *5*). *p < 0.05; **p < 0.01.

Since blood levels of MPA in women vary with initial dose and time following administration^34–37^, we performed a dose response study to more fully understand the range over which MPA suppresses wound closure. As seen in Fig. 4A, MPA at 10 and 100 nM significantly reduced wound closure by endometrial epithelial cells at 24 h post-injury but had no effect at 0.1 and 1 nM. NET and LNG had no effect on wound closure by endometrial epithelial cells at any concentration (0.1–100 nM) tested. We then extended our dose response studies to endometrial fibroblasts. Similar to epithelial cells, MPA at 10 and 100 nM suppressed wound closure of fibroblasts, while LNG had no effect at any dose tested (Fig. 4B). In contrast to epithelial cells, NET also suppressed wound closure of endometrial fibroblasts at 10 and 100 nM.

### Wound closure by endometrial epithelial cells and fibroblasts is not due to wound-stimulated cell proliferation

**Figure 5:**
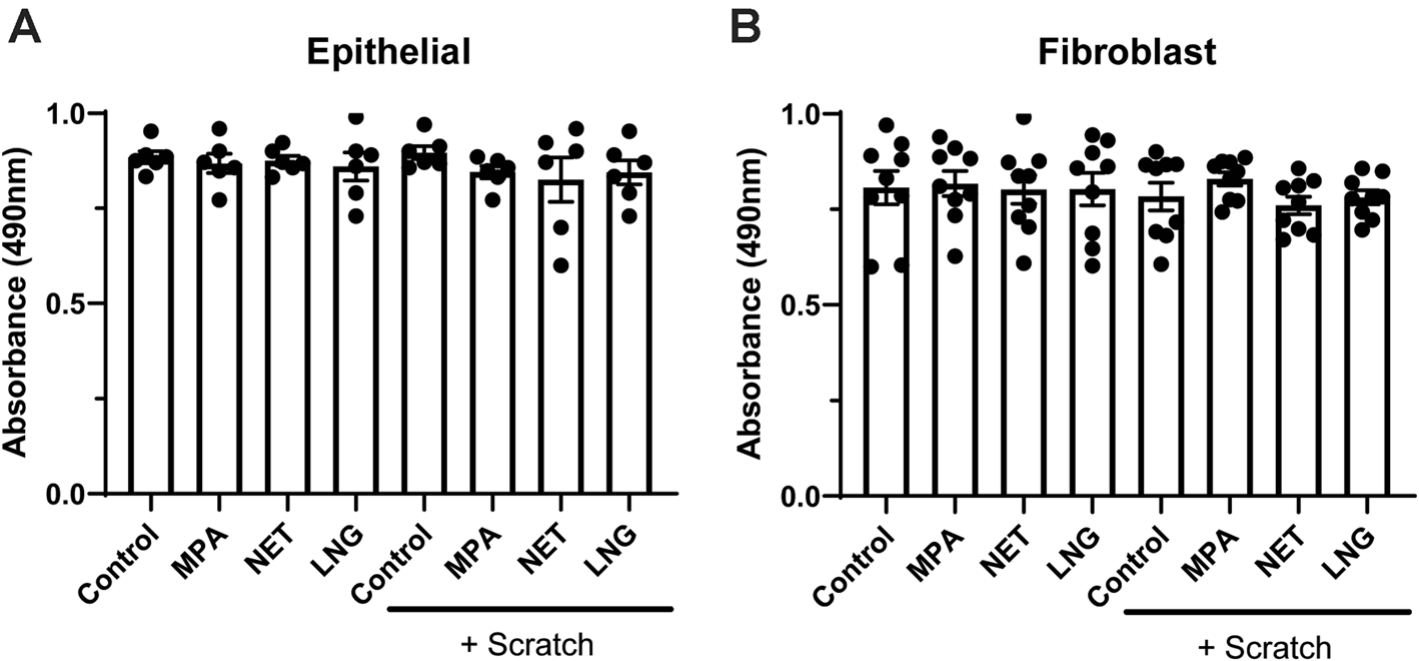
Wound closure is not accompanied by cell proliferation over 48 h. Cell proliferation following injury of **(A)** endometrial epithelial cells grown in transwell inserts and **(B)** endometrial stromal fibroblasts grown in 24-well plates. Cells were treated with MPA, NET, or LNG at 100 nM for 48 h prior to scratch, and maintained in cell culture media at 100 nM following scratch. Cell proliferation was determined using a CellTiter cell proliferation assay performed 48 h post-scratch. Bars represent mean + /− SEM. Each symbol represents a single individual (epithelial cell *n* = *6*; stromal fibroblast *n* = *9*).

Cell proliferation is an essential component of wound healing^38^. Given the rapidity of closure observed in our study, we asked whether endometrial epithelial or fibroblast wound closure was dependent on cell proliferation. We used the CellTiter cell proliferation assay to determine if MPA suppressed wound closure by epithelial cells and fibroblasts via decreased cell proliferation. As seen in Fig. 5, we measured cell proliferation in wounded and intact monolayers 24 h post-injury. We found that proliferation of epithelial cells and fibroblasts following injury was not significantly different from intact monolayers. Furthermore, we found that MPA had no effect on epithelial cell and fibroblast proliferation. Similarly, there was no effect of NET or LNG on proliferation by either cell type.

### MPA inhibits antimicrobial secretion by endometrial epithelial cells post-injury

**Figure 6:**
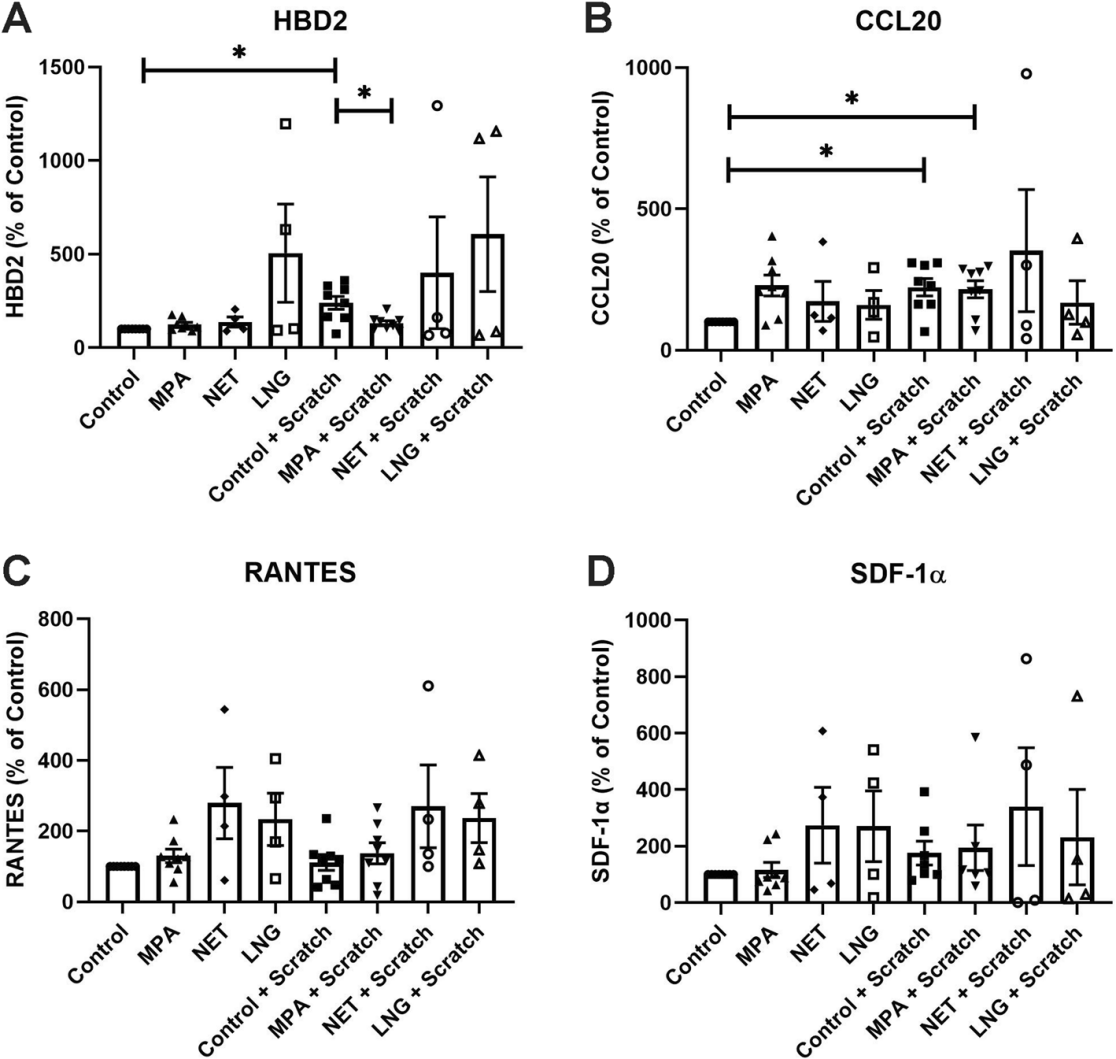
MPA inhibits the upregulation of HBD2 by polarized endometrial epithelial cells following injury. Secretion of **(A)** HBD2, **(B)** CCL20, **(C)** RANTES, and **(D)** SDF-1α by endometrial epithelial cells grown in transwell inserts 48 h post-scratch. Cells were treated for 48 h with 100 nM MPA, NET, and LNG prior to scratch and maintained in cell culture media following scratch. Bars represent mean + /− SEM. Secretion values were normalized to the control wells which were set to 100. Each symbol represents a single individual (Control *n* = *8*; MPA *n* = *8*; NET *n* = *4*; LNG; *n* = *4*). Mean secretion in controls samples: 850 pg/ml (HBD2), 575 pg/ml (CCL20), 37 pg/ml (RANTES), and 59 pg/ml (SDF-1α). *Control* untreated unscratched control, *Control + SC* scratched control, *MPA *unscratched MPA treated, *MPA + SC* scratched MPA treated, *NET* unscratched NET treated, *NET + SC* scratched NET treated, *LNG* unscratched LNG treated, *LNG + SC* scratched LNG treated. *p < 0.05.

Antimicrobials have an essential role in wound healing, acting as chemotactic proteins that induce the migration of immune cells to the wound site as well as protecting against pathogens at a potential site of entry. Using a multiplex assay developed by us^32^, we investigated whether progestins alter epithelial secretion of 4 proteins known to modulate wound healing at other sites: HBD2^39^, CCL20^39^, RANTES^40^, and SDF-1α^41^. As seen in Fig. 6, epithelial cells constitutively secreted all 4 proteins. Moreover, none of the progestins incubated with epithelial cells for 48 h post scratch had any effect on increased secretion relative to untreated controls. Unexpectedly, we found that MPA suppressed the injury-induced increase in HBD2 secretion by epithelial cells but had no effect on the secretion of CCL20, RANTES, and SDF-1α. NET and LNG had no effect on antimicrobial secretion by endometrial epithelial cells following injury compared to untreated controls. These results further demonstrate that injury stimulates the secretion of two key antimicrobials, HBD2 and CCL20, that play a role in wound closure and that MPA selectively suppresses the secretion of HBD2, suggesting a potential mechanism for delayed wound closure.

## Discussion

An intact mucosal barrier is essential for immune protection against incoming pathogens. However, mucosal surfaces are often damaged, and their rapid recovery from injury is necessary to reduce the risk of infection. Since STIs such as HIV can enter the underlying stromal tissue through breaches in the epithelium, it is crucial to understand the role of factors that influence the repair of the mucosal barrier following disruption. We demonstrate that MPA transiently inhibits wound closure by endometrial epithelial cells, while both MPA and NET inhibit wound closure by stromal fibroblasts in vitro. In contrast, LNG and progesterone had no effect on wound closure by either cell type. The suppressive effect of MPA on wound closure is not mediated by changes to cell proliferation, but probably by decreased cell migration in response to injury suggesting that within the first 24 h following injury, wound closure in our in vitro epithelial and fibroblast cultures is not driven by cell proliferation. During wound closure, the barrier function of epithelial cells exposed to MPA is transiently impaired, with decreased TER, suppression of HBD2 and increased flux of FITC-dextran across the epithelial monolayer. Thus, MPA disrupts the ability of endometrial epithelial cells and stromal fibroblasts to recover from injury, which could be a possible mechanism for increased risk of HIV/STI transmission in the upper FRT in vivo.

Similar to the lower FRT, the mucosal barrier in the endometrium and the rest of the upper FRT can undergo injury due to a variety of causes including ulcerations, endometritis, and cancer. In particular, endometritis is characterized by inflammation in the endometrium. Since inflammatory cytokines decrease TER of epithelial monolayers^42^, inflammation can lead to a weakening of the mucosal barrier. Several pathogens can cause endometritis, some of which such as *Neisseria gonorrhea* and *Chlamydia trachomatis* are also sexually transmitted. HIV also induces an inflammatory response that leads to decreased TER and increased translocation across the endometrial monolayer^4^. While sexual intercourse does not normally cause abrasive damage to the columnar epithelium of the upper FRT, in cases of cervical ectopy, where the columnar epithelium of the endocervix extrudes on to the surface of the ectocervix, sexual intercourse could lead to a damaged columnar epithelium. Therefore, it is important to recognize that in limited cases sexual intercourse can directly disrupt the columnar epithelium.

An important consideration for future studies is the to extent to which the negative effects of MPA on wound closure and barrier function are affected by the presence of other compounds such as antiretrovirals. Previously we used the same in vitro wound closure model to demonstrate that tenofovir, an antiretroviral used for pre-exposure prophylaxis, suppresses wound healing of endometrial epithelial cells and fibroblasts, at clinically relevant concentrations that prevent HIV transmission^31^. This suggested that beyond its anti-HIV effects, tenofovir may compromise wound healing, thereby extending the time of tissue immune cell exposure to HIV and other STIs. Our results extend these findings by demonstrating that the commonly used progestin MPA, which is linked with increased risk of HIV infection in women, also slows wound closure of human endometrial epithelial cells and stromal fibroblasts. While beyond the scope of our study, whether tenofovir and MPA have synergistic inhibitory effects on wound closure remains to be determined, but of importance given their combined widespread use in Sub-Saharan Africa. Beyond tenofovir and MPA, the question of whether other hormonal and drug compounds interact and affect wound closure and barrier integrity in the FRT is unclear. This has considerable clinical relevance since novel forms of multipurpose prevention technologies (MPT) such as vaginal rings and intra-uterine devices simultaneously deliver antiretrovirals and contraceptives into the FRT. Further studies are needed to understand how these compounds interact to affect barrier integrity and mucosal immune protection in the FRT.

Progestins have potent effects on the endometrium and their use is associated with breakthrough bleeding^43^, altered expression of basement membrane components, endometrial atrophy^44^, decreased number and size of epithelial glands, decreased stromal thickness, altered cytokeratin expression^45^, and decreased height of columnar epithelial cells^46^. Our demonstration that MPA slows wound closure by endometrial epithelial cells is consistent with those of previous studies that reported MPA inhibits migration of the Ishikawa endometrial epithelial cell line following injury via blockade of RANK/RANKL signaling, decreased vimentin expression, decreased IL-6 and TGFβ, and altered epithelial-mesenchymal transition^47,48^. We have extended these findings by demonstrating that MPA inhibits endometrial epithelial cell production of HBD2, which is important in wound repair at other mucosal surfaces. HBD2 signaling via CCR6 is essential for restitution by colonic epithelial cell lines by stimulating cell migration after injury^39^. Therefore, decreased expression of HBD2 may contribute to slower endometrial epithelial cell migration post-injury. CCL20, another CCR6 ligand, also stimulates wound healing by colonic epithelial cells^39^. Our finding that injury did not lead to an upregulation of CCL20, RANTES or SDF-1α secretion by endometrial epithelial cells, indicates that chemokines regulating epithelial cell migration are unique to distinct anatomical sites. Further studies are needed to understand the underlying mechanisms involved in wound closure in the upper FRT.

In addition to its role in epithelial wound repair, HBD2 is a potent inhibitor of pathogen survival. HBD2 has intrinsic antiviral and antibacterial activity and inhibits HIV infection of target cells in vitro^49^. Our findings of upregulation of HBD2 in response to epithelial injury suggests that, in addition to contributing to wound closure, HBD2 protects by preventing pathogens from infecting target cells in the underlying tissue. Since HBD2 production by injured epithelial cells exposed to MPA is suppressed, this suggests that antimicrobial protective responses are impaired, creating a more permissive environment for pathogen acquisition. This may represent an additional mechanism, beyond suppressing wound closure, by which MPA degrades immune function and increases the risk of HIV/STI transmission to women. Whether this occurs in vivo remains to be determined.

Beyond wound closure, MPA transiently inhibits the restoration of complete barrier function by endometrial epithelial cells, as demonstrated by decreased TER and increased FITC-dextran flux across the epithelial monolayer. The mechanisms behind this are unclear, but may involve changes to tight junction composition, which would permit increased movement from the apical to basolateral side of the epithelium. Our results are consistent with previous studies reporting that exposure to MPA in vivo leads to increased permeability of the intact vaginal, ectocervical, and endocervical epithelium to incoming pathogens or labelled particles in mice and macaques^50,51^ in the absence of injury. Similarly, MPA exposure in vitro decreased TER in polarized HEC-1A human endometrial epithelial carcinoma cells (0.38 mM)^52^ and primary endometrial epithelial cells (1 nM)^28^. In contrast to these results, we did not observe changes in TER and permeability across intact MPA-treated epithelial monolayers, but only following injury to MPA-treated monolayers. One reason for the differential observation in TER with intact MPA-treated monolayers could be due to the length of exposure to MPA. In contrast to previous in vitro studies, our study exposed epithelial cells to MPA for 48 h prior to injury and only after the cells had reached confluence and thus our results reflect an acute exposure to MPA. Dizzell et al.^28^ grew epithelial cells in MPA for at least 9 days before any significant differences were observed, while Irvin et al.^52^ used a higher dose of MPA (0.38 mM vs 100 nM). Overall, our studies with primary epithelial cells provide important new evidence that acute exposure to MPA can degrade barrier function following injury.

Intriguingly, there was differential sensitivity of epithelial cells and fibroblasts to progestins during wound closure: MPA inhibited wound healing by both epithelial cells and fibroblasts; P and LNG had no effect on epithelial cells or fibroblasts; and NET significantly inhibited wound closure by fibroblasts but not epithelial cells. The reasons for this remain unclear but are most likely related to the fact that all three compounds (MPA, NET and LNG) bind with varying affinity to the progesterone receptor (PR), glucocorticoid receptor (GR), and androgen receptor (AR)^53^. Signaling via the PR can affect wound closure. For example, in Ishikawa uterine epithelial cells, MPA signaling via the PR was necessary for inhibition of wound healing^48^. Since PR mRNA is present in both cultured endometrial epithelial cells and stromal fibroblasts, it is possible that MPA is exerting its suppressive effects in primary cells via the PR. However, we found no effect of progesterone on wound closure, suggesting that progesterone-mediated signaling via the PR is not sufficient for delayed wound closure. This does not imply that endometrial epithelial cells and fibroblasts are not responsive to progesterone as we have previously shown that endometrial epithelial cells increase their secretion of TGFβ following progesterone treatment^54^. Signaling via the PR is dependent upon PR protein levels, which in endometrial epithelial cells is upregulated following treatment with E_2_. Whether the lack of E_2_ in our system affects PR signaling due to lower PR expression is unknown, but of potential importance since many progestins are co-administered with E_2_. While PR mRNA was detectable in both epithelial cells and fibroblasts, we did not measure the PR protein levels. Whether PR mRNA levels are consistent with PR protein levels in our in vitro system is unknown and should be addressed in future studies.

An alternative explanation for the differential effects could be the ability of progestin compounds to bind to the GR and exert their effects via this pathway. Signaling via the GR can affect wound closure in multiple models. For example, dexamethasone, a potent specific GR agonist, inhibited wound healing by intestinal epithelial cell lines and co-cultures of gastric epithelial cells and fibroblasts^55,56^. Since MPA has a high affinity for the GR compared to the other progestins^53^, this difference may account for its inhibitory effect on epithelial cell and fibroblast wound closure. Given the varying affinity of progestins for each steroid hormone receptor, it is likely that multiple receptors are involved in wound healing. Whether the expression level of each steroid hormone receptor determines its contribution to wound healing in the presence of specific progestins is unknown. Future studies are needed to incorporate the responses of each cell type to specific progestins into an integrated model of wound healing that considers the contribution of each injured cell type and steroid hormone receptor.

An important consideration for in vitro studies is the appropriate dose of MPA. When administered as a contraceptive MPA has a dynamic concentration profile. Shortly after injection of 150 mg intramuscular MPA, serum concentration peaks at around 4.5–65 nM between 5 and 20 days^34^, before decreasing steadily and plateauing around 2.5 nM^35–37^ for the following three-month period. Karkkainen et al. demonstrated that MPA levels range from 1 to 35 nM over the first 24 h after administration^57^. Recently, Buckner et al. showed that plasma levels of MPA ranged from 10 to 100 nM^58^. To ensure that our study has biological relevance, we used concentrations of MPA (0.1, 1, 10, & 100 nM) that encompass the serum levels found in women using MPA as a contraceptive. We found the greatest effects on wound closure and barrier function at the higher concentration of 100 nM, but with effects on wound closure 10 nM. These doses (10 to 100 nM) are within the plasma concentration range in vivo for women using MPA as contraceptive particularly in the early period (days-weeks) following administration of MPA^7,58^, suggesting that MPA is capable of inhibiting wound closure in vivo.

Unlike MPA and NET, LNG had no effect on wound closure in either epithelial cells or fibroblasts. LNG may therefore be safer for immune protection, at least in terms of barrier function. This observation is consistent with clinical studies that suggest LNG use does not alter susceptibility to HIV transmission in women^17^. Importantly, the LNG concentration (100 nM) used in our study is comparable to that seen in the cervical secretions one month following LNG-IUD insertion^58^. This suggests that high concentrations of LNG in the FRT may not be detrimental to wound healing. However, it is unknown whether longer exposure to LNG would affect wound closure in our system. Since IUDs can be inserted for up to 5 years, further studies are required to determine whether long-term use of LNG could lead to impaired mucosal barrier function.

The columnar epithelium in the endometrium consists of both glandular and luminal epithelial cells, and our epithelial preparations consist of both populations. However, under our protocol we cannot differentiate between the two populations, though it is likely that the relative proportions isolated from each patient vary with age, menopausal status, and menstrual cycle stage. Others have shown that the luminal epithelium and glandular epithelium have unique and distinct phenotypic characteristics and have differential responses to hormonal stimuli^59^. Whether MPA, or the other progestins, have differential effects on wound closure or other phenotypic aspects of luminal and glandular epithelial cells is unknown and is an important consideration for future studies.

There are several important limitations to consider in our study. First, our wound closure model is exclusively in vitro, and therefore does not capture the complexity of an in vivo model in which multiple cell types are present. Second, we utilize monocultures of either epithelial cells or fibroblasts to investigate the effects of progestins. Whether the behavior of either epithelial cells or fibroblasts following injury, as well as the impact of MPA, changes in a mixed cell population either in vitro or in vivo is unclear. In vivo wound healing is the combined effect of multiple cell types acting in concert. In particular, immune cells which are essential for maximal wound healing in vivo are absent from our studies. Finally, we only studied the effect of MPA over a relatively short period of exposure. A single contraceptive dose of MPA can last for at least 3 months over which it has a varying concentration profile. Whether long-term exposure of epithelial cells and fibroblasts to MPA in vitro affects wound closure differently than short-term treatments is unclear. These questions should be addressed in future studies.

In conclusion, MPA slows wound closure by endometrial epithelial cells and fibroblasts. The delayed healing and altered immune responses in the presence of MPA potentially represent an avenue through which MPA increases the risk of HIV/STI transmission to women. Whether MPA also has detrimental effects on wound repair at other mucosal sites such as the anal and rectal epithelium is unknown, but of considerable importance given the increased risk of HIV/STI transmission following anal intercourse^60^. Beyond MPA, it is important to understand the effects of progestins and antiretrovirals, both alone and in combination, on mucosal surfaces and how this may impair the effectiveness of MPT such as microbicides, that are currently a major focus for HIV prevention.

## Supplementary Information

Supplementary Figure S1.
