# Supplementary material for: Medroxyprogesterone acetate inhibits wound closure of human endometrial epithelial cells and stromal fibroblasts in vitro
Source: Sci Rep. 2021 Dec 1;11:23246. doi: 10.1038/s41598-021-02681-6 (PMC8636475; doi:10.1038/s41598-021-02681-6)
Supplement: Supplementary file 1 — Supplementary Figure S1. [file 41598_2021_2681_MOESM1_ESM.docx]

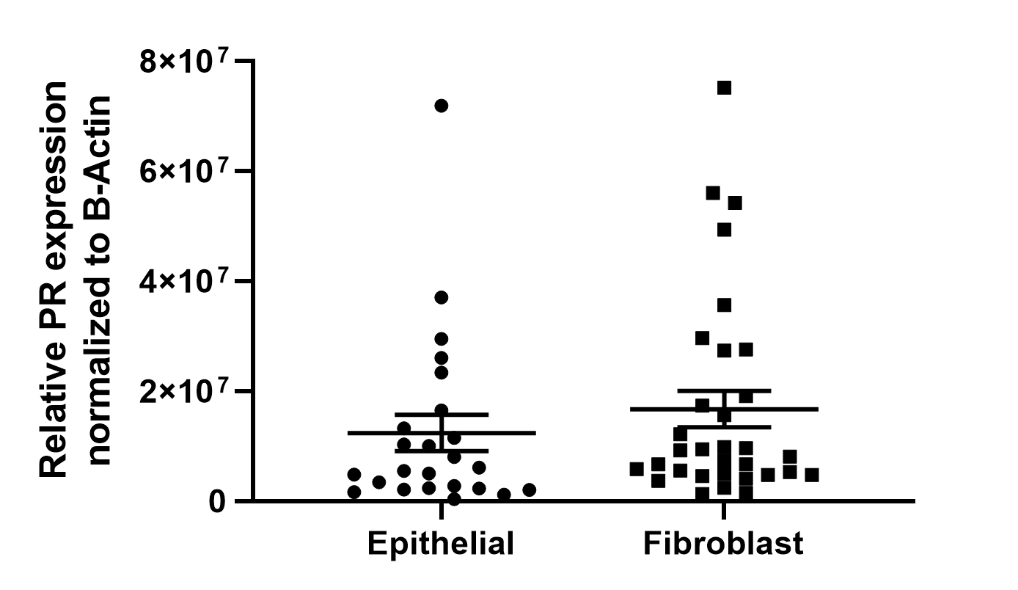


**Supplementary Figure 1**: Progesterone receptor (PR) mRNA expression was determined by real-time RT-PCR for endometrial epithelial cells (*n=24*) and endometrial stromal fibroblasts (*n=32*) grown *in vitro*.

Total mRNA was isolated and purified using a RNeasy mini kit (Qiagen, Valencia, CA) with on-column DNase digestion using the RNase-Free DNase set (Qiagen) according to the manufacturer’s recommendations. 400ng of total RNA was reverse-transcribed using the iScript cDNA synthesis kit (Bio-Rad) according to the manufacturer’s recommendations. Relative mRNA expression levels of PR (Hs01556702_m1) were measured using the 5' fluorogenic nuclease assay in real-time quantitative PCR using TaqMan chemistry on the ABI 7300 Prism real-time PCR instrument (Applied Biosystems, Carlsbad, CA). PCR was conducted using the following cycle parameters: 50°C, 2 mins, 1 cycle; 95°C, 10 mins, 1 cycle; 95°C, 15 s, 40 cycles; 60°C, 1 min, 1 cycle. Analysis was conducted using the sequence detection software supplied with the ABI 7300. Expression of PR is normalized to the expression of housekeeping gene β-Actin.
